# Percentage of the positive area of bone metastasis is an independent predictor of disease death in advanced prostate cancer

**DOI:** 10.1038/sj.bjc.6600715

**Published:** 2003-01-28

**Authors:** M Noguchi, H Kikuchi, M Ishibashi, S Noda

**Affiliations:** 1 Department of Urology, 67 Asahi-machi, Kurume University School of Medicine, Kurume, Fukuoka, Japan; 2 Division of Nuclear Medicine and Department of Radiology, 67 Asahi-machi, Kurume University School of Medicine, Kurume, Fukuoka, Japan

**Keywords:** prostate cancer, bone metastasis, bone scan

## Abstract

We addressed in this study whether quantifying the extent of disease on bone scans can predict the disease death of patients with advanced prostate cancer using computer-assisted image analysis. Pretreatment radionuclide bone scans were reviewed in 56 patients with bone metastases from prostate cancer, and the percentage of the positive area on a bone scan (%PABS) was quantified automatically using a personal computer with the NIH Image program for estimation of the accurate extent of metastatic bone lesions on a bone scan. The significance of the %PABS as well as the other known prognostic factors was evaluated using univariate and multivariate Cox proportional hazards analysis. In univariate regression analysis, the %PABS (*P*=0.0155), serum alkaline phosphatase (*P*=0.0272), the tumour grade based on biopsy (*P*=0.044) and the number of bone lesions on bone scans (*P*=0.0388) were well associated with disease-specific survival. In multivariate analysis, the %PABS (*P*=0.0155, relative risk ratio 2.603), but not the other factors, was the independent predictor of the disease death. These results suggest that the %PABS is a novel parameter for predicting the prognosis of patients with advanced prostatic cancer.

Bone metastasis is common in advanced prostate cancer and causes considerable morbidity including pain, pathological fractures and disability. The presence of bone metastasis is of prognostic importance in prostate cancer, because the extent of the disease (EOD) on a bone scan significantly affects overall survival (Soloway *et al*, 1988; Ernst *et al*, 1991). Bone scanning remains the most effective radiological procedure for detecting bone metastasis with 1% false-negative rate (Schaffer and Pendergrass, 1976), and for monitoring progression of bone metastasis. Visual analysis of bone scans is a common method to estimate the extent of skeletal disease. Several studies have proposed a grading classification for the bone metastasis based on the pattern of uptake on the bone scan (Amico *et al*, 1991; Chybowski *et al*, 1991) or based on the quantitation of the number of sites (Soloway *et al*, 1988; Erdi *et al*, 1997). However, quantitative analysis of all bone metastases is a time-consuming task because patients with advanced prostatic cancer usually have multiple disease sites. In addition, the assessment of response and progression largely vary, and thus it is difficult to have reproductive results in a large population through the conventional assessment. To more accurately determine the amount of tumour present at baseline and to monitor the tumour's response to therapy, we have addressed in this study whether quantifying the extent of bone metastases and express it as the percentage of the positive area on a bone scan (%PABS) can predict the disease death of patients with advanced prostatic cancer.

## Materials and methods

### Patients

We retrospectively reviewed 56 consecutive patients with prostate cancer with bone metastasis who visited the Kurume University Hospital in Japan from March 1994 to March 2000. All patients were newly diagnosed as histologically confirmed adenocarcinoma of the prostate, and none of them had previous treatment. Each patient was evaluated in a standard fashion with a careful history and physical examination, serum PSA determination, transrectal ultrasonography and radionuclide bone scan. All patients underwent computerised tomography (CT) or magnetic resonance imaging scan (MRI) of the pelvis. Serum samples were obtained and stored at −20°C. Serum PSA levels were determined using Tandem-R (Hybritech Inc., San Diego, CA, USA) assay with a normal range between 0 and 4.0 ng ml^−1^. The serum levels of pyridinoline crosslinked carboxyterminal telopeptide of type I collagen (ICTP) were measured using a two-antibody radioimmunoassay (RIA) using Telopeptide ICTP RIA kits (Orion Diagnostica, Espoo, Finland). ICTP is a peptide that is cleaved during type I collagen degradation and was suggested to be a marker of bone resorption (Risteli *et al*, 1993). The clinical usefulness of measuring the serum concentrations of ICTP as a marker for monitoring metastaic bone activity in patients with prostate cancer was reported elsewhere (Noguchi and Noda, 2001). Normal ranges of serum ICTP were 1.8–5.0 ng ml^−1^. Alkaline phosphatase activity in serum was measured using *p*-nitrophenyl phosphate substrate in diethanolamine buffer at 37°C with a normal range between 115 and 359 IU l^−1^. Tumour grade was determined according to the Gleason scale (Gleason, 1977), and classified as well-differentiated adenocarcinoma (Gleason score 2–4), moderate (Gleason score 5–7) and poor (Gleason score 8–10).

### Bone scintigraphy

Tchnetium-99m-hydorxy-methane-diphosphonate (HMDP) bone imaging was obtained after intravenous injection of Tc-99m HMDP (555MBq, Nihon Mediphysics Co. Ltd, Japan). Bone scintigraphy was obtained about 3 h after intravenous injection in all patients. Whole body images (scan speed 15 cm min^−1^, matrix 256×1024) were recorded with low energy, high-resolution collimator E.CAM, Siemens Medical Systems, Inc.). The whole body field was used to record anterior and posterior views digitally (256×1024) on a detected computSer system (Toshiba 5500A/PI, Tokyo, Japan). Energy discrimination was provided by a 10% window centred on the 140 keV of Tc-99 m. All scans were interpreted by one radiologist (MI) who was blinded to the patient's clinical condition, and the extent of metastatic bone disease was determined according to the number of bone lesions and the %PABS.

### Numeric counting

The total number of lesions was determined by visual counting of each discrete lesion using the method described previously (Soloway *et al*, 1988), and the metastatic findings on bone scans were classified into four groups in accordance with the EOD as follows: grade 1, less than six bone metastases (a lesion occupying the entire vertebral body was counted as two lesions); grade 2, 6–20 bone metastases; grade 3, more than 20 bone metastases, but less than a ‘super scan’ (diffuse symmetrical uptake without visualisation of the kidneys); and grade 4, super scan or its equivalent (involvement of greater than 75% of the ribs, vertebrae and pelvic bones). The total number of lesions on the ‘super scan’ was counted as 100 in this study.

### %PABS

**Figure 1:**
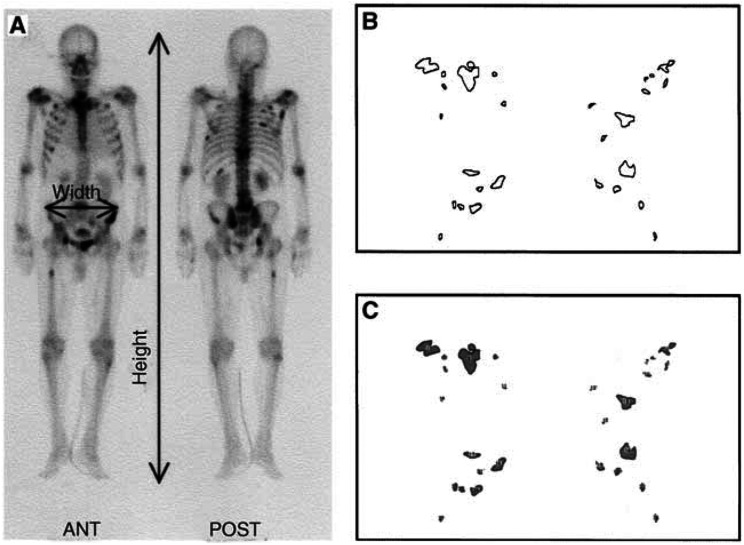
%PABS measurement. The %PABS is calculated using the formula %PABS=(positive area on bone scan/square area)×100. (**A**) The estimated square area is multiplied by the width at the gluteal region to the height of the entire skeleton on the bone scan. (**B**) Direct tracings of same hot spot perimeters in **B** from the bone scan. (**C**) Computer tracings after measurement by NIH Image.

To provide simply a quantitative measurement of the EOD, the bone scans were analysed according to the following criteria. All outlines of positive regions on a bone scan (both of anterior and posterior scans) were transferred by tracing to a comprehensive map of the entire bone metastasis. These findings were converted to image files using a digitising pad and a drawing program (MacPaint®, Claris Corp., Santa Clara, CA, USA) with a Macintosh computer (Apple, Inc., Cupertino, CA, USA) (Figure 1B

). The sum of all positive areas was automatically measured using an image analysis program (NIH Image, developed and maintained by the National Institutes of Health, Bethesda, MD, USA) (Figure 1C). The fractional involvement of the entire skeleton by metastasis was estimated as the %PABS. The %PABS was calculated using the formula %PABS=(positive area on bone scan/square area)×100, in which the square area was multiplied by the width at the gluteal region to the height of the entire skeleton on the bone scan (Figure 1A). The %PABS and number of bone lesions on five bone scans were measured by five recorders to compare the accuracy and time consumption of the %PABS method with the numeric counting. The coefficient of variation (CV) was calculated by dividing the sample s.d. by the sample mean. An average of five measurements were made for each bone scan.

After obtaining a written informed consent, all 56 patients were treated with luteinising hormone-releasing hormone agonist (either goserelin acetate of leuprolide acetate) and an antiandrogen agent (flutamide) or estramustine phosphate. Patients were clinically evaluated monthly for the first 3 months, and every 3 months thereafter. Biochemical tests and physical examination were repeated with the same frequency, while the chest film, bone scan and the other instrumental examinations were repeated every 6 months unless specific symptoms occurred.

### Statistical analysis

The baseline variables analysed in this study were age, Eastern Cooperative Oncology Group (ECOG) performance status, PSA, ICTP, alkaline phosphatase and %PABS. Correlation of the %PABS with number of bone lesions was evaluated by a mean of Pearson's correlation coefficient test. The disease-specific survival was determined by the Kaplan–Meier actuarial analysis, and the difference between the disease-specific survival and survival curve was assessed by the log-rank test. Cox proportional hazards regression model was used for univariate and multivariate analyses to identify factors that had a significant impact on survival. All baseline parameters in the survival and proportional hazards regression analysis were analysed as dichotomous variables using the overall median values as cutoff levels. All statistical calculations were carried out using the StatView® program (SAS Institute Inc., Cary, NC, USA). A value of *P*<0.05 was considered statistically significant.

## Results

In the initial analysis, the %PABS and number of bone lesions on five kinds of bone scans were measured, and the results are shown in Table 1

**Table 1:** Comparison between % PABS method and numeric counting

	**%PABS^a^**						**No. of bone lesions^b^**					
**Recorder**	**BS^c^-1**	**BS-2**	**BS-3**	**BS-4**	**BS-5**	**Measuring time (min)**	**BS-1**	**BS-2**	**BS-3**	**BS-4**	**BS-5**	**Measuring time (min)**
A	9.01	8.70	9.38	3.40	8.67	17	61	66	69	21	39	7
B	8.99	8.57	9.10	3.50	9.11	15	58	62	66	20	44	8
C	9.16	8.77	9.32	3.45	9.07	16	66	58	58	18	38	6
D	9.22	8.65	9.45	3.49	9.22	20	48	60	70	24	42	8
E	9.15	8.44	9.33	3.52	8.99	15	56	62	60	22	48	5
Mean	9.11	8.63	9.32	3.47	9.01	16.60	57.80	61.60	64.60	21.00	42.20	6.80
s.d.^d^	0.10	0.13	0.13	0.05	0.21	2.07	6.65	2.97	5.37	2.24	4.03	1.30
CV^e^ (%)	1.10	1.50	1.40	1.40	2.30	12.50	11.50	4.80	8.30	10.60	9.50	19.20

a NIH Image computerised measurement.

b Visually counting.

c Bone scintigraphy.

d Standard deviation.

e Coefficient of variation.

. The CV in the %PABS ranged from 1.0 to 2.3% (mean, 1.54%), while the numeric counting ranged from 4.8 to 11.5% (mean, 8.94%). The mean measuring time in both the %PABS and the numeric counting was 16.6 min (range, 15–20 min) and 6.8 min (range, 5–8 min), respectively.

The pretreatment clinical characteristics of the 56 patients are listed in Table 2

**Table 2:** Pretreatment clinical characteristics of the 56 study patients

		**EOD**			
	**Total**	**Grade 1**	**Grade 2**	**Grade 3**	**Grade 4**
No. of patient's	56	15	22	15	4
Patient's age (years)					
Mean/median	72/72	72/74	72/72	71/71	70/71
Range	49–93	53–93	59–88	49–88	65–75
EOCG performance status					
0+1	41	12	18	10	1
2+3	15	3	4	5	3
Biopsy grade					
Well differentiated	7	4	1	2	0
Moderately differentiated	27	9	10	7	1
Poorly differentiated	22	2	11	6	3
PSA (ngml^−1^)					
Mean/median	425/220	144/112	321/171	517/296	1713/2000
Range	2–2000	14–561	2–1755	19–1967	853–2000
Alkaline phosphatase (IUl^−1^)					
Mean/median	635/467	343/233	499/357	1001/718	1101/774
Range	91–4751	118–1079	194–2334	91–4751	659–2197
ICTP (ngml^−1^)					
Mean/median	8.5/6.1	5.1/4.5	7.1/5.9	11.9/6.6	16.9/17.1
Range	1.9–52	2.5–8.5	1.9–26.5	4.3–52	11.2–22.2
No of bone lesions					
Mean/median	23/14	4/4	13/12	38/30	100/100
Range	1–100	1–5	6–20	21–80	100–100
PABS (%)					
Mean/median	7.7/4.6	1.6/0.9	4.0/3.6	14.1/12.8	26.7/23.3
Range	0.2–43.3	0.2–5.9	1.5–9.7	501–27.6	17.1–43.3

**Figure 2:**
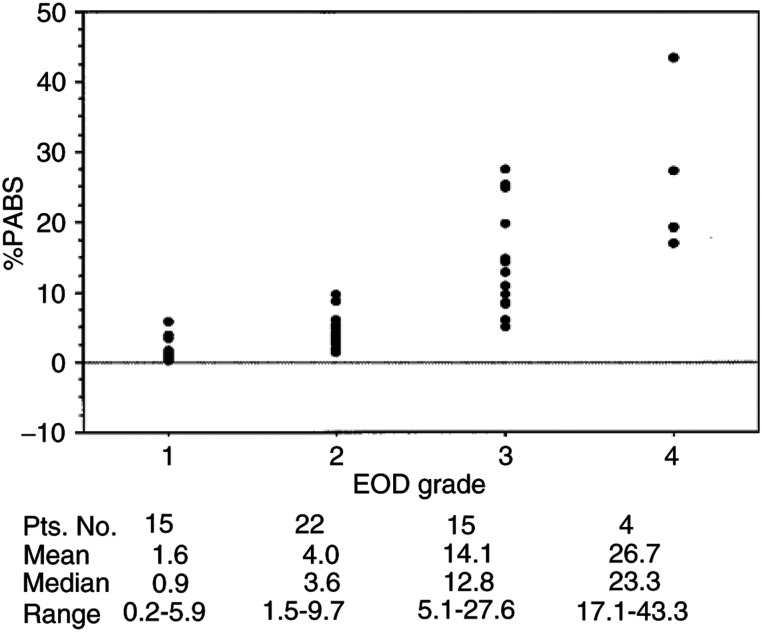
Correlation between the %PABS and the number of bone lesions.

**Figure 3:**
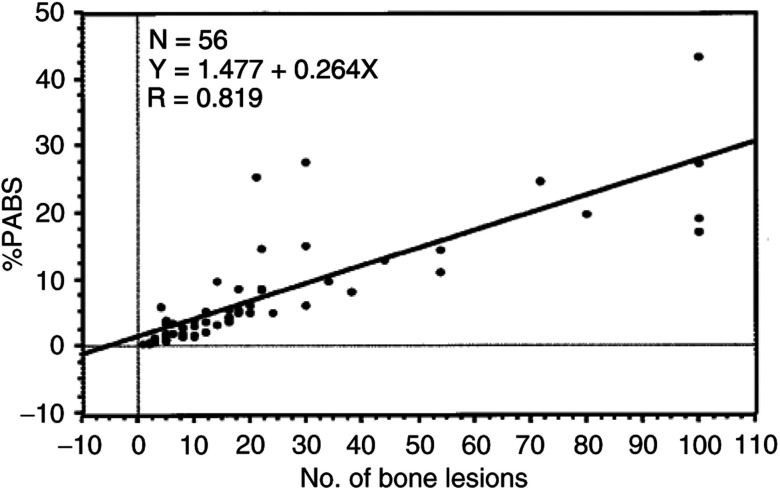
Correlation between the %PABS and the EOD grade from 1 to 4.

. The median age of the patient population was 72 years. A median of bone lesions per patient was 14. Of the 56 cases, 22 (39%) had EOD grade of 2, 15 (27%) had grade 1, 15 (27%) grade 3 and only 4 (7%) grade 4. The median values for PSA, alkaline phosphatase and ICTP increased with the extent of bone metastasis. Figure 2

shows the linear regression analysis among the %PABS measured using the NIH Image computer program and by visual counting of the number of bone lesions. The %PABS well correlated with the number of bone lesions with a higher (*R*=0.819 and *R*^2^=0.671; *P*<0.001) correlation in grade 1 and 2 patients (*R*=0.732 and *R*^2^=0.536, *P*<0.001). The median %PABS for the prostate cancer patients with bone metastasis sharply increased as EOD grade progressed as follows: 0.9 for grade 1, 3.6 for grade 2, 12.8 for grade 3 and 23.3 for grade 4. Although %PABS well correlated with EOD grade when examined as groups, there were many overlaps in grading the bone metastasis in individual patients (Figure 3

).

**Figure 4:**
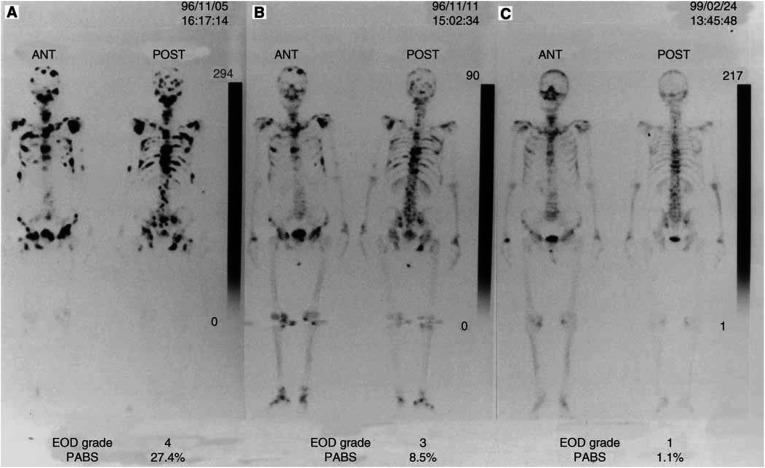
A serial bone scan in a patient who showed improvement of bone metastasis after hormonal therapy: (**A**) pretreatment; (**B**) 12 months after treatment; (**C**) 27 months after treatment.

A serial bone scan in a patient who showed improvement of bone metastasis after hormonal therapy is shown in Figure 4

. The initial involvement in the bone slowly improved between 5 November 1996 and 24 February 1999 with a marked decrease in %PABS. However, it was difficult to count the accurate number of multi bone lesions using the visual counting.

All patients received a hormonal therapy with various kinds of regimen. The hormonal therapy included combined androgen blockade (leuprolide plus flutamide or bicalutamide) (27 patients), leuprolide plus estramustine phosphate (24 patients), flutamide alone (three patients) and leuprolide alone (two patients). The mean length of follow-up for all patients was 32 months (median, 29; range, 4–86), and 28 patients (50%) died because of prostate cancer. The mean time to disease death was 27 months (median, 25; range, 4–50). The mean follow-up for those patients who did not die because of prostate cancer was 36 months (median, 33; range, 4–86). None of the patients were lost to follow-up during this analysis. Only three out of eight patients who died of diseases other than prostate cancer had an observation time shorter than 24 months (4, 10 and 12 months). Cox proportional hazards regression analysis was performed to determine factors that would predict the disease death ( Table 3

**Table 3:** Cox proportional hazards regression analysis for 56 patients with prostate cancer with bone metastasis

		**Univariate**			**Multivariate**	
**Variable**	**Cutoffs**^a^	***P*-value**	**Relative risk ratio**	**95% CI**^b^	***P*-value**	**Relative risk ratio**
%PABS (%)	4.6 or greater/less than 4.6	0.016	2.603	1.199–5.651	0.016	2.603
Serum alkaline phosphatase (IUl^−1^)	467 or greater/less than 467	0.027	2.452	1.016–5.434	—	—
No. of bone lesions	14 or greater/less than 14	0.044	2.222	1.024–4.824	—	—
Tumour grade on biopsy	Poorly/well or moderately	0.044	2.153	1.021–4.540	—	—
Serum ICTP (ngml^−1^)	6.1 or greater/less than 6.1	0.103	1.906	0.878–4.136	—	—
EOD grade	3 or 4/1or2	0.104	1.868	0.879–3.970	—	—
Patient's age (years)	72 or greater/less than 72	0.326	1.462	0.685–3.119	—	—
Serum PSA (ngml^−1^)	220 or greater/less than 220	0.361	1.430	0.664–3.079	—	—
EOCG performance status	1 or 2 or 3/0	0.474	1.426	0.540–3.767	—	—

a %PABS, alkaline phosphatase, number of bone lesions, ICTP, patient age and PSA are based on median values, and the remaining are treated as dichotomous variables.

b Confidence intervals. Of the 56 patients, 28 (50%) had cancer death.

). In univariate regression analysis, %PABS (*P*=0.0155), serum alkaline phosphatase (*P*=0.0272), the tumour grade based on biopsy (*P*=0.044) and the number of bone lesions on bone scans (*P*=0.0388) were significantly associated with the disease-specific survival. Any of the others studied (age, performance status, pretreatment PSA and bone metabolic markers) were not significant factors. Forward stepwise, multivariate analysis showed that only %PABS (*P*=0.0155, relative risk ratio 2.603) was an independent predictor of the disease death. Patients with less than 4.6 %PABS had a significantly better disease-specific survival compared with those with greater than 4.6 %PABS (*P*=0.011, Figure 5

**Figure 5:**
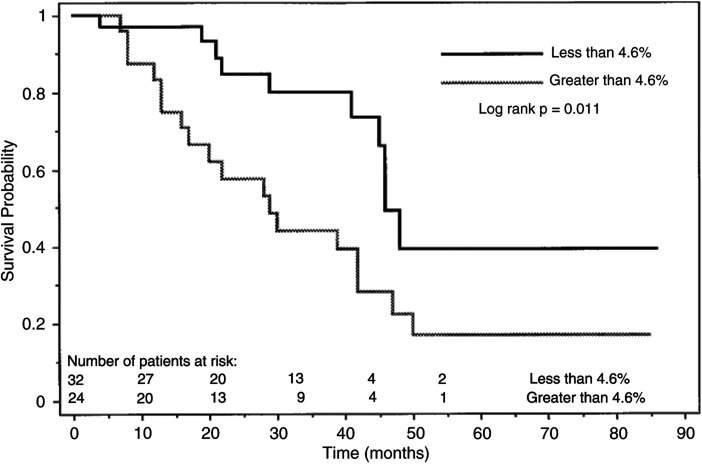
Kaplan–Meier plot shows disease-specific survival after treatment of metastatic prostate cancer for those with less than 4.6 %PABS and greater than 4.6 %PABS (*P*=0.011).

). All other variables in Table 3 were insignificant predictors of the disease death after hormonal treatment.

## Discussion

Radionuclide bone scans are strongly positive in cases of bone involvement of prostate cancer patients, irrespective of whether the lesions are radiologically lytic, mixed or pure blastic (Citrin *et al*, 1981). The conventional method to monitor the response of bone metastases to therapy is a combining qualitative assessment of sequential bone scans and bony films with measurement of chemical markers (such as PSA and alkaline phosphatase level). However, to quantitate all bone metastases in patients is a time-consuming task, since patients with metastatic involvement usually have more than one disease site. Several studies have evaluated different ways to quantify the extent of bone involvement during therapy (Table 4

**Table 4:** Definitions of grading systems for the extent of skeletal disease in prostate cancer using a bone scan

**Reference**	**Method**	**Description**
Soloway *et al* (1988)	Visual inspection	The total number of bone lesions were counted visually and graded into five categories: 0, normal; 1, less than six lesions; 2, 6–20 lesions; 3, more than 20 lesions but not a superscan; 4, superscan.
Amico *et al* (1991)	Visual inspection	A bone scan scoring system from 0 to 2, 0 represents normal uptake, 1 means one or several uptakes and 2 signifies diffuse uptake.
Chybowski *et al* (1991)	Visual inspection	A simple bone scan scoring system was similer to the above. This clasification was negative, positive or indeterminate.
Dann *et al* (1987)	Measurements of the total radiotracer uptake	The 24-h whole body retention was measured as an objective marker for bone metastasis.
Sabbatini *et al* (1999)	Visual inspection	A BSI was based on the known proportional weights of each of the 158 bones that were derived from the reference man.
Erdi *et al* (1997)	Computer analysis	The images were transferred to a computer for ROI analysis and the BSI was calculated.
Present study	Visual and computer analysis	The %PABS was quantified automatically using a computer with the NIH Image.

). The EOD was graded into five categories by visually counting the total number of bone lesions on the bone scan (Soloway *et al*, 1988). This method has the difficulty of counting individual lesions when lesions increase in number. It was also recognised that errors would be inevitable because lesions in the ribs are difficult to count and quantify, and assessment of lesions in the pelvis is complicated by the three-dimensional nature of the pelvic bone. In the present study, there was a weak correlation between the %PABS measured using the NIH computer program and the counted number of bone lesions in patients with EOD grades 3 and 4 (greater than 21 bone metastases) compared with grades 1 and 2 (less than 21 bone metastases). Other studies have proposed staging classifications based on visual inspection, whether the lesions on the bone scan occurred with normal uptake, several uptakes or diffuse uptake (Amico *et al*, 1991), or according to the negative *versus* positive (Chybowski *et al*, 1991). These simplified grading systems showed significant associations with survival (Crawford *et al*, 1989; Rana *et al*, 1993). It is, however, difficult to evaluate serial changes on bone scans after treatment, because the criteria for response and progression vary widely. A simple and reproducible technique was evaluated to measure the 24-h whole body retention (skeletal uptakes) as an objective marker for bony metastases in comparison to clinical outcome (Dann *et al*, 1987). This method could be easily automated, but much useful information such as the anatomical information, about which bones are involved or progressing, was lost in this simplified approach. As a result, none of these methods were adopted into routine clinical practice. Some studies have developed a bone scan index (BSI) to more accurately quantify the extent of the skeletal involvement by the tumour. This BSI method is based on a subjective interpretation of the bone scan, in which the fraction of each bone involved is visually estimated (Imbriaco *et al*, 1998; Scabbatini *et al*, 1999). This technique is a complex method to calculate the sum of the involved area in the 158 bones by summing the fractions times the percentage of the skeleton for each bone, and has shown a variation from 0 to 50% among individual studies in the estimations. Techniques that use computer generated regions of interest (ROI) for regional bone uptake have been attempted in the BSI method (Erdi *et al*, 1997). However, expense, long processing times and confusion relating to the exact region to choose made this technique less than ideal.

In the present study, we used the NIH Image program for measurements of all positive areas on bone scans transferred by manual tracing to a comprehensive map. This method showed a good accuracy for area measurements with a 1.54% CV. The main advantages of the %PABS method are both its simplicity and accuracy in measurements of ROI. However, although these measures correlate with the extent of bone metastases in patients with prostate cancer, the prognostic significance of these measurements has not previously been demonstrated.

The conventional pretreatment risk factors for advanced prostatic cancers were performance status, the presence or absence of bone pain, tumour grade, biochemical parameters such as haemoglobin, alkaline phosphatase and testosterone levels and the EOD grade (Emrich *et al*, 1985; Soloway *et al*, 1988; Chodak *et al*, 1991). PSA is the most widely used tumour marker for diagnosis and follow-up of prostate cancer, but the importance of pretreatment PSA in predicting outcome after therapies was not yet confirmed. In this study, we investigated the relation between survival time and seven potential prognostic factors (age, performance status, tumour grade, PSA, alkaline phosphatase, ICTP and %PABS), using univariate and multivariate regression models. The tumour grade, %PABS, alkaline phosphatase and number of bone lesions were derived from a univariate analysis of prognostic factors for the survival in this series, although the tumour grade, alkaline phosphatase and the number of bone lesions did not remain independent of prognostic value by multivariate analysis. Only the %PABS was a significant predictor of survival after hormonal treatments. Performance status, which was reported as a very strong independent parameter previously (Chodak *et al*, 1991), was not a significant predictor. One possible reason for this discrepancy might be in part because of heterogeneity of treatment in the present study. Although the total number of patients in this study was small, the number of patients who had disease death (26 patients, 50%) was quite high, and makes the significance of the data from this study strong relative to its size.

## Conclusions

The findings of the present study suggest that the %PABS quantified automatically using the NIH Image program is a simple and reproducible estimate of the percentage of the skeleton involving tumours in patients with advanced prostate cancer. This method may be useful to stratify patients in clinical trials and may provide prognostic information. A large-scaled trial with more statistical power is needed to demonstrate that %PABS in patients with prostate cancer with bone metastasis is a significant predictor of survival after hormonal treatments. Further studies are required to assess the utility of serial %PABS determination in monitoring treatment effects.

## References

[bib1] Amico S, Liehn JC, Desoize B, Larbre H, Deltour G, Valeyre J (1991) Comparison of phosphate isoenzymes PAP and PSA with bone scan in patients with prostate carcinoma. Clin Nucl Med 16: 643–648 1718651 10.1097/00003072-199109000-00006

[bib2] Chodak GW, Vogelzang NJ, Caplan RJ, Soloway M, Smith JA (1991) Independent prognostic factors in patients with metastatic (stage D2) prostate cancer: The zoladex study group. JAMA 265: 618–621 1824790

[bib3] Chybowski FM, Keller JL, Bergstralh EJ, Oesterling JE (1991) Predicting radionuclide bone scan findings in patients with newly diagnosed untreated prostate cancer: prostate specific antigen is superior to all other clinical parameters. J Urol 145: 313–318 1703240 10.1016/s0022-5347(17)38325-8

[bib4] Citrin DL, Cohen AI, Harberg J, Schlise S, Hougen C, Benson R (1981) Systemic treatment of advanced prostatic cancer: development of a new system for defining response. J Urol 125: 224–227 6259376 10.1016/s0022-5347(17)54980-0

[bib5] Crawford ED, Eisenberger MA, McLeod DG, Spaulding JT, Benson R, Dorr DG, Blumenstein BA, Davis MA, Goodman PJ (1989) A controlled trial of leuprolide with and without flutamide in prostatic carcinoma. N Engl J Med 321: 419–424 2503724 10.1056/NEJM198908173210702

[bib6] Dann J, Castronovo Jr FP, McKusick KA, Griffin PP, Strauss HW, Pout Jr GR (1987) Total bone uptake in management of metastatic carcinoma of the prostate. J Urol 137: 444–448 3102758 10.1016/s0022-5347(17)44062-6

[bib7] Emrich LJ, Priore RL, Murphy GP, Brady MF, and the investigators of the national prostatic cancer project (1985) Prognostic factors in patients with advanced stage prostate cancer. Cancer Res 45: 5173–5179 4027993

[bib8] Erdi YU, Humm JL, Imbrico M, Yeung H, Larson M (1997) Quantitative bone metastases analysis based on image segmentation. J Nucl Med 38: 1401–1406 9293797

[bib9] Ernst DS, Hanson J, Venner PM (1991) Analysis of prognostic factors in men with metastatic prostate cancer. Uro-Oncology Group of Northern Alberta. J Urol 146: 372–376 1856934 10.1016/s0022-5347(17)37797-2

[bib10] Gleason DF (1977) Histologic grading and clinical staging of carcinoma of the prostate. In Urologic Pathology, The Prostate. Tannenbaum M (ed) pp 171–197. Philadelphia, PA: Lea & Febiger

[bib11] Imbriaco M, Larson SM, Yeung HW, Mawlawi OR, Erdi Y, Venkatraman ES, Scher I (1998) A new parameter for measuring metastatic bone involvement by prostate cancer: the bone scan index. Clin Cancer Res 4: 1765–1772 9676853

[bib13] Noguchi M, Noda S (2001) Pyridinoline cross-linked carboxyterminal telopeptide of type I collagen as a useful marker for monitoring metastatic bone activity in men with prostate cancer. J Urol 166: 1106–1110 11490307

[bib14] Rana A, Chisholm GD, Khan M, Sekharjit SS, Merrik MV, Elton RA (1993) Patterns of bone metastasis and their prognostic significance in patients with carcinoma of the prostate. Brit J Urol 72: 933–936 8306158 10.1111/j.1464-410x.1993.tb16301.x

[bib15] Risteli J, Elomana I, Niemi S, Novamo A, Risteli L (1993) Radioimmunoassay for the pyridinoline cross-linked carboxyterminal telopeptide of type I collagen: a new serum marker of bone collagen degradation. Clin Chem 39: 635–640 8472358

[bib16] Scabbatini P, Larson SM, Kremer A, Zhang ZF, Sun M, Yeung H, Imbriaco M, Horak I, Conolly M, Ding C, Ouyang P, Kelly WK, Scher H (1999) Prognostic significance of extent of disease in bone in patients with androgen-independent prostate cancer. J Clin Oncol 17: 948–957 10071289 10.1200/JCO.1999.17.3.948

[bib17] Schaffer DL, Pendergrass HP (1976) Comparison of enzyme, clinical, radiographic, and radionuclide methods of detecting bone metastases from carcinoma of the prostate. Radiology 121: 431–434 981622 10.1148/121.2.431

[bib18] Soloway MS, Hardeman SW, Hickey D, Raymond J, Todd B, Soloway S, Moinuddin M (1988) Stratification of patients with metastatic prostate cancer based on extent of disease on initial scan. Cancer 61: 195–202 3334948 10.1002/1097-0142(19880101)61:1<195::aid-cncr2820610133>3.0.co;2-y

